# Research on predicting the driving forces of digital transformation in Chinese media companies based on machine learning

**DOI:** 10.1038/s41598-024-57873-7

**Published:** 2024-03-27

**Authors:** Zhan Wang, Yao Li, Xu Zhao, Yuxuan Wang, Zihan Xiao

**Affiliations:** 1 College of Humanities and Communication, Dongbei University of Finance and Economics, Dalian, China; 2 School of Information Science and Technology, Dalian University of Science and Technology, Dalian, China; 3 Surrey International Institute, Dongbei University of Finance and Economics, Dalian, 116025 Liaoning China

**Keywords:** Digital transformation, Machine learning, Chinese media companies, Media economics, Information technology, Socioeconomic scenarios, Sustainability

## Abstract

Chinese media companies are facing opportunities and challenges brought about by digital transformation. Media economics takes the evaluation of the business results of media companies as the main research topic. However, overcoming the internal differences in the industry and comprehensively predicting the digital transformation of Chinese media companies from multiple dimensions has become an important issue to be understood. Based on the “TOE-I” theoretical framework, this study innovatively uses machine learning methods to predict the digital transformation of Chinese media companies and to analyze specific modes of the main driving factors affecting the digital transformation, using data from China’s A-share-listed media companies from 2010 to 2020. The study found that environmental drivers can most effectively and accurately predict the digital transformation of Chinese media companies. Therefore, under sustained and stable economic and financial policies, guiding inter-industry competition and providing balanced digital infrastructure conditions are keys to bridging internal barriers in the media industry and promoting digital transformation. In the process of transformation from traditional content to digital production, media companies should focus on policy changes, economic benefits, the decision-making role of core managers, and the training and preservation of digital technology talent.

## Introduction

In the digital economy era, media has become ubiquitous in our lives. With the help of digital technologies such as mobile Internet, big data, cloud computing, the Internet of Things (IoT) and artificial intelligence, companies involved in the media industry are facing new development opportunities. The 14th Five-Year Plan (2021–2025) in the National Economic and Social Development and Vision 2035 of the People’s Republic of China lists digital transformation as a national development strategy. This plan clearly outlines the implementation of the digital cultural industry, in accelerating the development of new cultural enterprises, cultural forms, and cultural consumption patterns, and strengthening digital creativity, network audio-visuals, digital publishing, digital entertainment, and the online broadcast industry. COVID-19 has had a serious negative impact on China’s economic and social development. However, driven by digital technology, the traditional media industry pattern has undergone fundamental changes, and the digital transformation and development of media has become an inevitable trend^1^. In 2022, China’s media industry output fell 2.11% at a value of 290,825 billion yuan, presenting an overall decline but a local rise. Internet advertising, Internet marketing services, mobile data and Internet business, online games and other traditional high-output areas show different degrees of negative growth. The total revenue of radio and television advertising, book sales, and newspaper industry was less than that of the network audio-visual field^2^.

Although media companies are part of the media industry, their driving forces and the pressures of digital transformation differ due to their different businesses. China’s media industry has certain ideological attributes, especially political attachments represented in publishing, radio, and television mainstream media^3,4^. Based on guidance promoting the amalgamation of traditional and emerging media in 2014, the Chinese government provides huge external policy support for media companies whose main businesses are in publishing, radio, and television. Advertising and film industries have also been affected by the digital transformation from business processes to organizations and business models. Hence, it is necessary to understand how to overcome the internal differences in the industry and make a comprehensive evaluation and prediction of the digital transformation of Chinese media companies.

Existing research has largely focused on the correlation between single-dimension characteristics and media digital transformation, only making predictions within a sample. There is little comprehensive consideration of the driving forces in the digital transformation of media companies^5–7^. This study is based on the theory of TOE (Technology-Organization-Environment), taking the media industry category as an important dimension in predicting the digital transformation of media companies, and building an out-of-sample prediction model for digital transformation (i.e. a “TOE-I” model) of media companies from the dimensions of technology, organization, environment, and media industry classifications. We aim to analyze the differences in the prediction ability of digital transformation behavior of different types of elements in varying dimensions and to identify the main factors and patterns of influence that drive media companies to participate in digital transformation. Our highlight can be divided into two aspects: (1) the advanced empirical research methods based on machine learning, and (2) the innovative theoretical framework which is applicable to the prediction of Chinese media companies’ digital transformation.

This research innovatively adopted the ensemble learning method in machine learning to explore the main cause behind the digital transformation of Chinese media companies. The advantages of this method are as follows: Firstly, the existing literature regarding media companies’ digital motivation has tended to use multiple linear regression, which cannot accurately reflect the complex relationship between variables due to the nonlinear characteristics of normal relationships. In contrast, ensemble learning can effectively handle the nonlinear relationship and possible interactions between variables; thus the resulting model can better reflect the information. Furthermore, the ensemble learning method does not need to preset the functional form of variables in the model but fits the relationship between variables as much as possible according to the training set data. This is more suitable for predictive analysis than traditional linear research methods^8^. Secondly, ensemble learning can make use of relative importance and partial dependence plots and other means to analyze different variables on the prediction ability of digital behavior, and can describe the specific variables affecting the data transformation of media companies to facilitate comparative analysis between variables. Thirdly, although machine learning methods are applied more in the field of natural sciences than in social science^9^, the advanced learning power and self-correction ability of machine learning are suitable for quantitative analysis of causal relationships between economic variables. They can also produce more accurate estimates of control variables, such as fixed effects outside the sample. Therefore, compared to traditional multiple linear regression, machine learning has the advantages of flexibility, accuracy, and foresight.

The media has undergone significant changes in a short period, due to technological changes. From publishing, broadcasting, and television, to the digital platform-based Internet and marketing, there is a significant gap between the business of companies in the media industry and the production of content and products. As mentioned earlier, the media industry represented by radio, television, and newspapers has strong political attributes that differ from the advertising, marketing, and gaming industries that actively integrate into international capital operations and look at the global market. Therefore, following existing literature practices^10–12^, this article innovatively divides the driving forces of the digital transformation of Chinese media companies into the following four dimensions of driving forces (adding “I” to “TOE”): (1) Technical, (2) Organizational, (3) Environmental, and (4) Industrial. This article explores the factors that drive or hinder the digital transformation of media companies from these perspectives. Based on the research results, corresponding policy implications are proposed.

This study is based on the “TOE-I” framework. Part 1 is the introduction of our research and in Part 2 we conduct the literature review mainly focused on the application of machine learning in media economics and the basic “TOE” framework. Part 3 is the methodology including the research design, data sources and variable definitions and empirical results and analysis. Part 4 is the conclusion of our main findings and we provide suggestions for policy makers and companies.

## Literature review

### The influencing factors of enterprise digital transformation

Current relevant research into enterprises’ digital transformation largely focuses on two perspectives: the driving and hindering factors of digital transformation, and the impact of digital transformation on all aspects of the enterprise. Regarding the drivers of digital transformation, Verhoef et al.^13^ focus on the response of enterprises to the changes in digital technology, increased digital competition, and the consequential digitalized customer behavior. While emerging digital technologies reduce labor costs, competition among companies intensifies and consumer preferences change accordingly. Yan et al.^14^ found that mixed ownership reform is the catalyst for digital transformation and is also the key driving force for the sustainable development of China’s state-owned enterprises. In addition, digitalization has become an important factor affecting the decision-making processes of entrepreneurs, and digital strategy is an important part of the corporate strategy of enterprises^15^. Regarding the factors hindering the digital transformation of enterprises, Roman and Rusu^16^ found that a lack of technology and capital negatively influences the digital transformation of enterprises, and that digital infrastructure has become an external factor affecting the digital transformation of enterprises. Looking at the impact of digital transformation, there is a focus on the relationship between digital transformation and the performance of enterprises^17^. Driven by the pursuit of profits, digital transformation has promoted the financialization of enterprises, especially among companies with poor internal and external governance^18^. The digital transformation of enterprises significantly promotes mergers and acquisitions by reducing internal organizational costs and is more significant among private enterprises^19^.

There are also many studies on digital transformation in different industries and different types of enterprises. For instance, Lange et al.^20^ collected data from semi-structured interviews and concluded capital to be a driver for digital start-ups in massive and rapid business scaling (MRBS). Roman and Rusu^16^ established an econometric-based model and highlighted the relationship between the performance of SMEs and digital transformation indicators. Ardolino et al.^21^ focused on how digital capabilities (IoT, cloud computing, and predictive analytics) support the service transformation of industrial enterprises.

### Application of machine learning in the field of media economics

Media economics is a discipline at the intersection of economics, management, and communication. It has shifted from the traditional media industry represented by the printing, television, and film industries to the new media research period with the Internet, digital platforms, and mobile communication media as the main focus^22^. The study of media economics under the corporate paradigm, and the evaluation of the operational results of media organizations has always been an issue. To evaluate business performance, Huang^23^ put forward an evaluation system to measure the financialization level of a media company, including the index of the ownership structure, shareholder value, financial asset holding ratio, and financial investment rate. Sheng et al.^24^ established an evaluation system on the performance of media organizations’ mergers and acquisitions to evaluate the value-creation ability after mergers and acquisitions. Xie and Li^5^ looked at the evaluation of the competitiveness of listed media companies during the big data era.

At the core of artificial intelligence technology, machine learning technology has been widely used in the field of journalism and communication; for example, in the mode reformation of content production^25,26^, the prediction and discovery of social media trends, and the emotional analysis of users^27–29^. These methods are occasionally used in predicting and analyzing the operation and development of media companies. Pan and Wang^6^ used machine learning methods to conduct text analysis of the annual report information of media companies to identify the relationship between digital transformation and the value of cultural enterprises. Shi and Wang^30^ focused on the advertising industry, combining artificial neural network (ANN) algorithms to achieve intelligent evaluation and predictive analysis of advertising publishing and click results, and to optimize the resource utilization efficiency of the advertising industry. Sun et al.^31^ used text mining and natural language processing (NLP) technology to conduct an emotional analysis on negative reports on the operation and financial status of media companies and established a warning mechanism for adverse impact on financial status.

In summary, this study has found that in the field of media economics, research is focused on macro perspective industry characteristics such as the operation and management of integrated media and the development and operation models of new media formats. From the perspective of micro market entities, however, there is still a lack of theoretical and empirical research on the transmission path to the industry change brought by digital transformation within media companies. Analysis and research on the driving factors of the implementation of digital transformation strategies in media companies through the predictive ability of machine learning is rare. This research aims to find indicators to measure the degree of digital transformation in the media industry and applies the machine learning method to identify the key elements driving the digital transformation of the media industry in China.

### The “TOE-I” prediction model

The theoretical framework of TOE (Technology, Organization, Environment) was first proposed by Tornatzky and Fleischer to comprehensively study and analyze the influencing factors that may cause interference when enterprises adopt innovative technologies^32^. At the technology level, the TOE framework considers the influence of the internal technical level and technical support-related factors within an organization; that is, whether the enterprise can apply existing technologies, which is the basis for enterprises to adopt innovative technology^33,34^. At the organizational level, to achieve the future application of innovative technologies within the organization, the focus is on the composition of specialties and responsibilities of organizational personnel at different levels^35,36^. Environmental factors represent the macro external characteristics of the specific environment where the organization operates, such as government policies^37^, competitive pressure, and the business environment^38^. The TOE framework systematically considers technology and organizational factors both inside and outside organizations so it has strong systematization and operability.

However, industrial segmentation in different industries faces various digital transformation challenges in the digital era. Some scholars have proposed that to determine the specific factors in these three backgrounds and establish the potential relationships between these factors, the TOE framework serves as a basic framework to integrate other relative elements^33^. Influenced by technological changes, media has changed dramatically in a short period of time. From publishing to radio and television then to the Internet and marketing on a digital platform, there is a big gap between the business and the produced content of the media industry. Therefore, based on the characteristics of Chinese media companies, this study takes “I”-Industry as an important dimension to predict their digital transformation and integrates it into the TOE framework.

Based on existing literature practices^10–12^, this study divides the driving force of the digital transformation of Chinese media companies into the following four dimensions: (1) Technical driving force. Digital technology is the basis of the digitalization of the media industry and should be applied in all fields, particularly the production and operation process of the media industry^10,12^. The technical upgrading of enterprises is directly reflected in the investment in technical research and development and in the scale of technical personnel^39,40^. (2) Organizational driving force. The heterogeneity of corporate internal governance subjects, such as the characteristics of senior executives, enterprise organization, and governance structure will lead to different behavior in digital transformation among media companies^41^. Based on previous research^6,7^, the characteristics of the organizational driving force include the size, knowledge level, and social resources of the senior management team, as well as the revenue ability, debt repayment ability and continuous growth ability. (3) Environmental driving force. In the tide of media globalization, Chinese media companies represented by advertising and games expand their overseas markets, while media companies such as radio, television, and publishing (combining social and corporate attributes) are influenced by policy. Therefore, this study includes the opening rate of monetary policy, financial support, and competition pressure in the industry, as well as the level of protection of intellectual property by local government as environmental driving factors. (4) Industrial driving force. Publishing, radio and television, advertising and film, games and digital media face different industry bases and characteristics in the digital transformation. Mainstream media, represented by publishing and radio and television, actively develop new forms and content based on new media platforms. They also undertake political tasks in guiding public opinion and “narrating Chinese stories well”^3,4^. Big data and intelligent algorithms have continued to erode the boundaries of the traditional advertising industry, causing collective concerns in the advertising industry^42^. Within the industrial driving force dimension, China’s listed media companies are subdivided into six industries of games, advertising marketing, film and television cinema, digital media, publishing, and television broadcasting in predicting the driving force of the industry.

This article therefore aims to use the TOE model and takes “Industry” as one of the influencing factors based on the particularity of the Chinese media industry to explain why Chinese media companies conduct digital transformation, thereby filling the theoretical gap in the interaction mechanism between companies and industry characteristics in the Chinese media industry. To obtain a more accurate model, the study chose machine learning methods. Based on the practices of other research, we innovatively used ensemble learning models other than text analysis, such as Random Forest Regression (RFR) and Gradient Boosting Regression (GBR) models to expand the application of machine learning methods in the media field.

## Method

### Research design

#### Research methods

This study uses an integrated machine learning method to construct and integrate multiple base learners to achieve more accurate prediction effects than using a single one. According to the degree of independence among the base learners, the method of Nie et al.^43^ and Parzinger et al.^44^ selected the Gradient Boosting Regression (GBR) and Random Forest Regression (RFR) in serial and parallelization methods, then compared them with multiple linear regression and LASSO in a linear research method. The integrated machine learning method effectively illustrates the non-linear relationships and interactions between the variables in the linear relationship, so that it performs well in out-of-sample prediction tasks ^8^. Therefore, this study predicts that integrated machine learning methods will outperform linear research methods in predicting the degree of digital transformation of media companies.

#### Model design

The model performance is examined from two perspectives: the model interpretation ability and the prediction error. In terms of model interpretation ability, Chen et al.^45^ and Ghazwani and Begum^46^ illustrate that ensemble learning can adjust itself based on the deviation between the model fitting value and the observation value in the previous calculation and can self-check the accuracy of the model. Therefore, we believe that the difference between the estimated values of the model and the observations can be used as a standard to evaluate the interpretation ability of the prediction model. The following two indicators are used: (1) Intra-sample goodness of fit (${\text{R}}_{\text{Is}}^{2}$) to evaluate the fitting effect of each research method on the training set sample. With the higher within-sample goodness of fit, the model is also more interpretable to the training set samples. (2) Out-of-sample goodness of fit (${\text{R}}_{\text{oos}}^{2}$) to measure the universality of the model. In addition, this study measures the generalization ability of the model from the perspective of variance, and chooses the explanatory variance to measure the dispersion of the actual value $\text{(}{\text{EVS}}_{\text{oos}}$).

In terms of model prediction error, we followed the practice of Chen et al.^47^ in selecting the out-of-sample mean variance (${\text{MSE}}_{\text{oos}}$) to investigate the deviation degree between the predicted and actual value. The out-of-sample mean square error is positively correlated with the accuracy of the model prediction. To avoid the large deviation value in the test set, which leads to estimated mean square error inconsistency, the average absolute error (${\text{MAE}}_{\text{oos}}$) and absolute median $({\text{MedAE}}_{\text{oos}})$ differences were used to evaluate the accuracy of the model prediction. The specific methods of each evaluation index are shown in Table 1.

**Table 1:** Model evaluation indicators and calculation methods.

Evaluation indicators	Indicator description	Equation
${\text{R}}_{\text{Is}}^{2}$	Intra-sample goodness of fit; in the training set, the model predicts values to the observed values	${\text{R}}_{\text{Is}}^{2}/{\text{R}}_{\text{oos}}^{2}\text{=1-}\frac{{\sum }_{\text{i=1}}^{\text{n}}{\left({\text{y}}_{\text{i}}-\widehat{{\text{y}}_{\text{i}}}\right)}^{2}}{{\sum }_{\text{i=1}}^{\text{n}}{\left({\text{y}}_{\text{i}}-\stackrel{\mathrm{-}}{\text{y}}\right)}^{2}}$
${\text{R}}_{\text{oos}}^{2}$	Out-of-sample goodness of fit; in the training set, the model predicts values to the observed values	
${\text{EVS}}_{\text{oos}}$	Interpretable variance; in the prediction set, the model predicts the degree of fit to the variation of the observed value	${\text{EVS}}_{\text{oos}}\text{=1-}\left({\text{var}}\left(\text{y} - \widehat{\text{y}}\right)\right)/\left({\text{var}}\left({\text{y}}\right)\right)$
${\text{MSE}}_{\text{oos}}$	Mean squared error; the expected value of the square between the out-of-sample predicted value and the actual value	${\text{MSE}}_{\text{oos}}\text{=}{1}/{\text{n}}{\sum }_{\text{i=1}}^{\text{n}}{\left({\text{y}}_{\text{i}}-\widehat{{\text{y}}_{\text{i}}}\right)}^{2}$
${\text{MAE}}_{\text{oos}}$	Average absolute error; the expected value of the difference between the out-of-sample predicted and actual value	${\text{MAE}}_{\text{oos}}\text{=}{1}/{\text{n}}{\sum }_{\text{i=1}}^{\text{n}}{\left|{\text{y}}_{\text{i}}-\widehat{{\text{y}}_{\text{i}}}\right|}^{2}$
${\text{MedAE}}_{\text{oos}}$	Absolute median difference, the median of the absolute difference between out-of-sample predicted and actual values	${\text{MedAE}}_{\text{oos}}= \text{median of } \left|{\text{y}}_{\text{i}}-\widehat{{\text{y}}_{\text{i}}}\right|$

In interpreting the model results, the integrated machine learning method includes multiple learners so it cannot be directly explained as much as a single learner^48^. To solve this problem, we used a relative importance and partial dependency graph to interpret the practical significance in the ensemble machine learning model. Relative importance refers to the degree of importance of one variable relative to the others in the process of model fitting. According to the method of Supsermpol et al.^49^, the relative importance of the variable can be assessed by measuring the decrease of the variable after its introduction. If the relative importance of a variable is high, it has a stronger influence in predicting the digital transformation of media companies. The partial dependency graph illustrates measurement of the influence of the changing degree of a certain variable on the digital transformation of a media company, assuming that other features are unchanged. Moreover, it is displayed in the form of images, which have more visual features. The single variable is more accurate in predicting the degree of digital transformation of media companies^50^.

### Data sources and variable definitions

#### Data source

This study selected media companies listed on A-shares in 2010–2020 as the initial sample, with company data from Wind and CSMAR databases. To exclude the interference of any special observation samples to the prediction results, the data were processed as follows: (1) exclusion of enterprises with ST, PT, and other abnormal listing status to avoid interference with the overall prediction effect due to abnormal operation of the enterprise itself; (2) elimination of samples with missing data; and (3) continuous variables in the data were treated by 1% and 99% quantile to avoid extreme outlier interference. A final set of 395 observations were obtained. The classification of the media industry adopts the 2021 SHENYIN&WANGUO classification method in the CSMAR database.

#### Variable definition

The digital transformation index (Digitaltransindex) in the CSMAR database was selected as the response variable. According to the CSMAR variable, the response variable using the annual report of enterprise digital transformation-related word frequency statistics can effectively reflect the enterprise digital transformation and transformation degree. It is divided into five parts: artificial intelligence (AI), blockchain (BD), cloud computing (CC), big data (BD) and the application of digital technology level (ADT). Table 2 shows the detailed calculations.

**Table 2:** Response variable definition.

Type of variable	Variable name	Variable definition
Y	Digitaltransindex	CSMAR digital transformation index in the database

**Figure 1:**
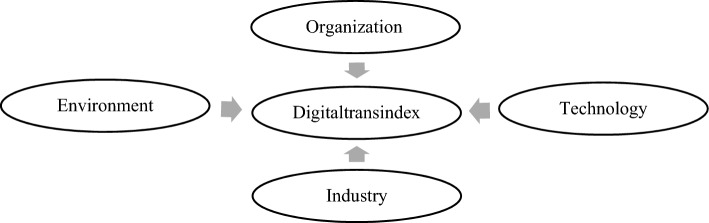
Four dimensions in TOE-I model.

Based on the existing research of digital transformation drivers, this study selected the driving force characteristics of the model from the following four dimensions, as shown in Fig. 1:

This study draws on Yang and Xu^39^ and Li^9^ to select the intensity of R&D expenses and the proportion of technical personnel as the measurement indicators of innovation ability and absorption ability, as shown in Table 3.

**Table 3:** Technical variable definitions.

Type of variable	Variable name	Variable definition
Technology	R&D expenses	R&D investment intensity (ratio of R&D investment to operating income)
	Technical size	The proportion of technical personnel (ratio of technical personnel to total employees)

In terms of organizational dimensions, there are two main factors that affect the strategic decisions in a company’s digital transformation. The first is the leadership style of the company’s CEO and management. The attitude of the executive team towards risk, as well as the decision-making style and decision-making power of the management are closely related to the implementation level of the digital transformation strategy. The second factor lies in the internal operations and cash flow of the company. The implementation of digital transformation strategy requires a large amount of capital participation so the use and fundraising of internal and external funds of enterprises should be listed as influencing factors. Referring to Bernile et al.^51^, Schoar and Zuo^52^, and Bandiera et al.^53^, Manager Number, Education Level, Social Network, ROA, Growth, TobinQ, Lev, Top Ten Holders’ Rate, Duality, and IndDirector Ratio were selected as these variables^51–53^. Detailed calculations are shown in Table 4.

**Table 4:** Organizational variable definitions.

Type of variable	Variable name	Variable definition
Organization	Manager number	Natural logarithm of the total number of managers
	Education level	The education level of the senior executive team is measured, that is, the value of other degrees is 1, the college degree is 2, 3, and the graduate degree is 4. The sum of the weight of the senior executive team is divided by the total number of people to obtain the average number of the education level of the senior executive team
	Social network	The total number of senior executives working in other enterprises in the corresponding year
	Top ten holders’ rate	Share ratio of the top ten shareholders
	Duality	Duality = 1, non-duality = 0
	IndDirector ratio	The proportion of the number of independent directors to the number of the board of directors
	ROA	Return on assets ROA. (Profit for the year/total assets)
	Lev	The ratio of total liabilities to total assets
	Growth	(Operating income of this year/operating income of last year) − 1
	TobinQ	(Market value of tradable shares + number of non-tradable shares net assets per share + book value of liabilities)/total assets

Also, with reference to Xu et al.^54^, Sun and Zheng^55^, Wu and Ma^56^, this study took Financial Support, Infrastructure Score, Monetary Policy, IP Protection, and HhiD as variables to measure the environmental characteristics of media companies. The above indicators reflect the overall business environment of the media industry and the support from governments in different regions for innovative development in the media industry, as shown in Table 5.

**Table 5:** Environmental variable definition.

Type of variable	Variable name	Variable definition
Environment	Financial support	The ratio of the local financial expenditure on science and technology to the public budget revenue
	Infrastructure score	The entropy right method is used to construct the infrastructure application and development indicators supporting the development of digital economy into an infrastructure index, with provincial annual data
	Monetary policy	The annual M2 growth rate for that year
	IP protection	The ratio of the contract amount of the technology market of each province to the GDP of each province in the current year is divided into provincial annual data
	HhiD	The Herfindahl–Hirschman Index of the industry

The fourth dimension is the industry classification. According to the revised version of the SHENYIN&WANGUO classification 2021, the media industry is subdivided into six categories: games, advertising and marketing, film and television cinema, digital media, publishing, and TV broadcasting, with a total of 141 listed companies, as shown in Table 6.

**Table 6:** Industry variable definitions.

Type of variable	Variable name	Variable definition
Industry category	Industry name	According to the revised version of SHENYIN&WANGUO classification 2021, the media industry is subdivided into six categories of games, advertising marketing, film and television cinema, digital media, publishing, and TV & radio

Similarly, this study draws on Li et al.^57,58^, Zhao et al.^59^, Hanelt et al.^60^ in taking Past Revenue, Cash Flow Ratio, Firm Age, Firm Size, and SOE as benchmark variable groups, as shown in Table 7.

**Table 7:** Benchmark variable definitions.

Type of variable	Variable name	Variable definition
Benchmark variable	Past revenue	Natural logarithm of company revenue at the end of the year
	Cash flow ratio	Operating net cash flow / total assets
	Firm age	Company listing years
	Size	Log of the total assets
	SOE	Soes = 1, non-soes = 0

### Empirical results and analysis

#### Descriptive statistics

As shown in Table 8, the mean value of the digital transformation index of media companies is 493.9037975, and the standard deviation is 297.7080399, indicating that the degree of digital transformation differs significantly among industries, and the characteristics of other variables have no outliers.

**Table 8:** Descriptive statistics.

	Count	Mean	Std	Min	25%	50%	75%	Max
Index	395	493.9038	297.708	4	242.5	466	689.5	1059
Year	395	2016.939	2.439388	2011	2015	2017	2019	2020
Group	395	0.632911	0.482622	0	0	1	1	1
R&D expenses	395	5.679929	7.779755	0	0.88	3.0042	7.155	71.85
Lev	395	0.330351	0.159494	0.052611	0.201259	0.314267	0.417194	0.934305
Top ten holders rate	395	60.83413	14.47212	22.32	50.87	62.06	71.725	92.53
Growth	395	0.335457	0.598806	− 0.97998	0.003664	0.224988	0.490722	3.941812
Past revenue	395	21.4649	1.172927	18.88874	20.55911	21.50047	22.26976	24.42523
Cash flow ratio	395	0.079033	0.091225	− 0.35322	0.030374	0.071103	0.117397	0.635785
Size	395	22.25187	1.055435	19.5677	21.46037	22.19776	23.10506	24.52307
Manager number	395	1.729422	0.33833	0	1.609438	1.791759	1.94591	2.70805
SOE	395	0.349367	0.477375	0	0	0	1	1
Technical size	395	5.680981	7.779339	0	0.88	3.01	7.155	71.85
Financial support	395	4.252152	0.165679	3.98	4.13	4.25	4.41	4.49
Monetary policy	395	10.53874	2.122946	8.275	8.375	10.32667	12.32	14.84667
HhiD	395	0.151643	0.126422	0.004297	0.045287	0.112874	0.231362	0.578315
TobinQ	395	2.291807	2.060475	0.837022	1.320861	1.74079	2.526945	31.40024
ROA	395	0.03161	0.120481	-0.70856	0.024287	0.049672	0.076015	0.384027
Firm age	395	16.67342	5.918411	4	12	16	20	39
IndDirector ratio	395	38.32489	5.3177	28.57	33.33	37.5	42.86	60
Education level	395	3.376421	0.304143	2.25	3.218254	3.416667	3.580128	4
Social network	395	18.13418	4.864637	10	15	17	20	45
Duality	395	0.63038	0.483314	0	0	1	1	1
IP protection	395	0.037309	0.055471	0.000591	0.006273	0.012114	0.027716	0.17495
Infrastructure score	395	0.201656	0.055325	0.108821	0.158169	0.19676	0.234819	0.479432
Digitaltransindex	395	45.42496	8.738175	24.7246	39.6917	46.9371	51.5884	65.4063
AD	395	0.207595	0.406099	0	0	0	0	1
Digitalmedia	395	0.086076	0.280832	0	0	0	0	1
Film	395	0.106329	0.308649	0	0	0	0	1
Game	395	0.281013	0.450064	0	0	0	1	1
Publish	395	0.189873	0.392698	0	0	0	0	1
TV & radio	395	0.129114	0.335751	0	0	0	0	1

#### The prediction effect of the model constructed based on the machine learning method on the digital transformation index of media companies

Table 9 shows the prediction results of the models constructed by different integrated machine learning methods on the degree of digital transformation of media companies. The results in column (1) show that the within-sample goodness of fit of the multiple linear regression and LASSO model is lower than that of the GBR and RFR, indicating that the within-sample fitting effect of the integrated learning method is superior. In addition, the results of columns (2) and (3) in Table 9 show that the out-of-sample goodness of fit and interpretable variance of GBR have the highest values, 0.59123809 and 0.55601084, respectively, followed by RFR. Four indicators of both methods are higher than 0.5, indicating that machine learning methods can better predict the degree of digital transformation of media companies. It is clear that in column (4) the out-of-sample mean square error of the GBR and the RFR is smaller than the multiple linear regression and LASSO. Finally, columns (5) and (6) indicate that the GBR and RFR have low mean absolute errors, 0.57720771 and 0.58604578, respectively. This indicates that the model improvement effect is not obvious after excluding the deviation value.

**Table 9:** Results of model fitting.

	${\text{R}}_{\text{Is}}^{2}$ (1)	${\text{R}}_{\text{oos}}^{2}$ (2)	${\text{EVS}}_{\text{oos}}$ (3)	${\text{MSE}}_{\text{oos}}$ (4)	${\text{MAE}}_{\text{oos}}$ (5)	${\text{MedAE}}_{\text{oos}}$ (6)
Multiple linear regression	0.41842795	0.21139459	0.21402806	0.8353831	1.18867211	0.64738668
LASSO	0.05334293	0.01292332	0.02660003	0.90533417	1.48782967	0.66869436
GBR	0.90395376	0.59123809	0.59546335	0.57720771	0.61613055	0.42558157
RFR	0.92743321	0.55325681	0.55601084	0.58604578	0.67338008	0.42271498

In conclusion, the GBR and RFR in the ensemble machine learning method fit better to the data, thus constructing a more accurate prediction research model. This study further discusses the driving forces of the digital transformation of media companies and the key factors.

#### Differences in the driving force dimensions of media companies' digital transformation prediction ability

To explore the different driving force dimensions of media company digital transformation prediction ability, this study first constructed the listed years (Firm Age) and company size (Size) using control characteristics such as benchmark model calculation and comparison to add different driving force combinations of prediction performance. As the research conclusions obtained based on different evaluation indicators are largely the same, this study analyzes the out-of-sample goodness of fit and the research results are as shown in Table 10.

**Table 10:** Prediction performance under different combinations of driving forces.

${\text{R}}_{\text{oos}}^{2}$	Multiple linear regression (1)	LASSO (2)	GBR (3)	RFR (4)
Benchmark	0.101436	0.031870	0.081122	0.166972
Benchmark + technology	0.105532	0.031870	0.271218	0.321959
Benchmark + organization	0.264684	0.117570	0.267906	0.334702
Benchmark + environment	0.203275	0.039836	0.462744	0.409237
Benchmark + industry	0.202921	0.031870	0.325166	0.380639
Benchmark + technology + organization	0.269030	0.117571	0.354623	0.391439
Benchmark + technology + environment	0.204882	0.039836	0.532292	0.438514
Benchmark + technology + industry	0.241420	0.031870	0.420074	0.475880
Benchmark + technology + environment	0.301312	0.136863	0.370195	0.459327
Benchmark + organization + industry	0.314237	0.117570	0.368417	0.415728
Benchmark + environment + industry	0.289889	0.039836	0.539554	0.508942
Benchmark + technology + organization + environment	0.303901	0.136863	0.423259	0.434396
Benchmark + technology + organization + industry	0.326098	0.117571	0.409223	0.427079
Benchmark + technology + environment + industry	0.323807	0.039836	0.551842	0.522093
Benchmark + organization + environment + industry	0.368127	0.136863	0.457802	0.451701
Benchmark + technology + organization + environment + industry	0.379480	0.136863	0.466834	0.474714

Firstly, we considered the difference in the ability of single-dimensional drivers to predict the digital transformation of media companies. In comparison with other driving forces, the addition of environmental driving force features to the benchmark model achieves the best prediction effect. Taking RFR as an example, after adding technology, organization, environment, and industry drivers to the benchmark model, the predicted value increased by 92.86%, 100.53%, 145.17%, and 128.04%, respectively. Secondly, we considered differences in the ability of different combinations of drivers to predict the digital transformation of media companies. A combination including environmental driving force dimensions has the best performance: when the two types of driving force characteristics are combined, the benchmark model adds the environmental driving force and the industry driving force can obtain a higher model interpretation ability. When the technology driving force, environmental driving force and industry driving force are added to the benchmark model, the highest model interpretation ability is achieved. The results show that the environment driving force is more accurate in predicting the digital transformation degree, indicating that stable monetary policy, comprehensive infrastructure construction, government financial support for digital transformation, and good industry concentration are key elements in driving the digital transformation of Chinese media companies.

#### Differential analysis of the prediction ability of digital transformation by key factors under different driving forces

**Figure 2:**
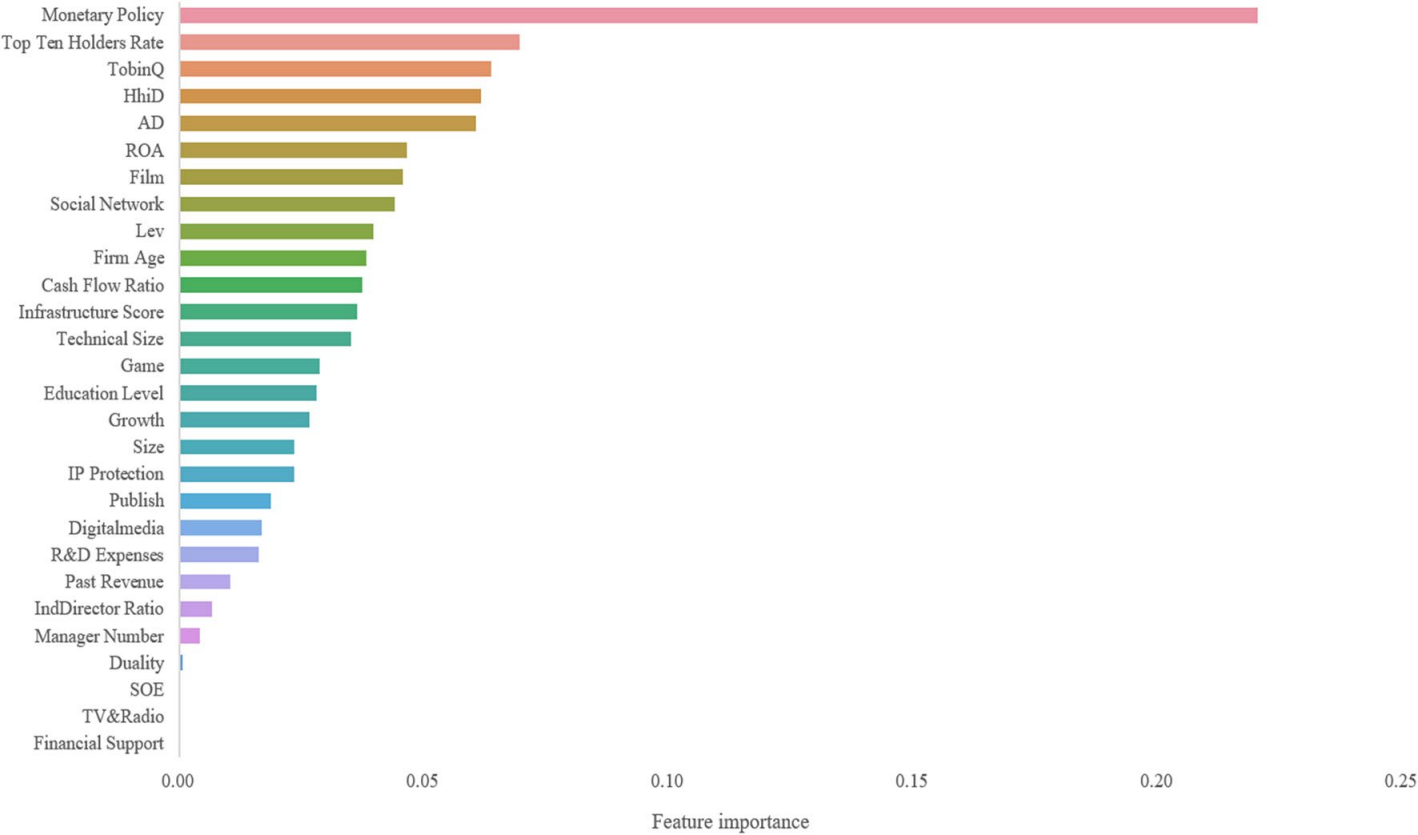
Relative importance ranking based on GBR.

**Figure 3:**
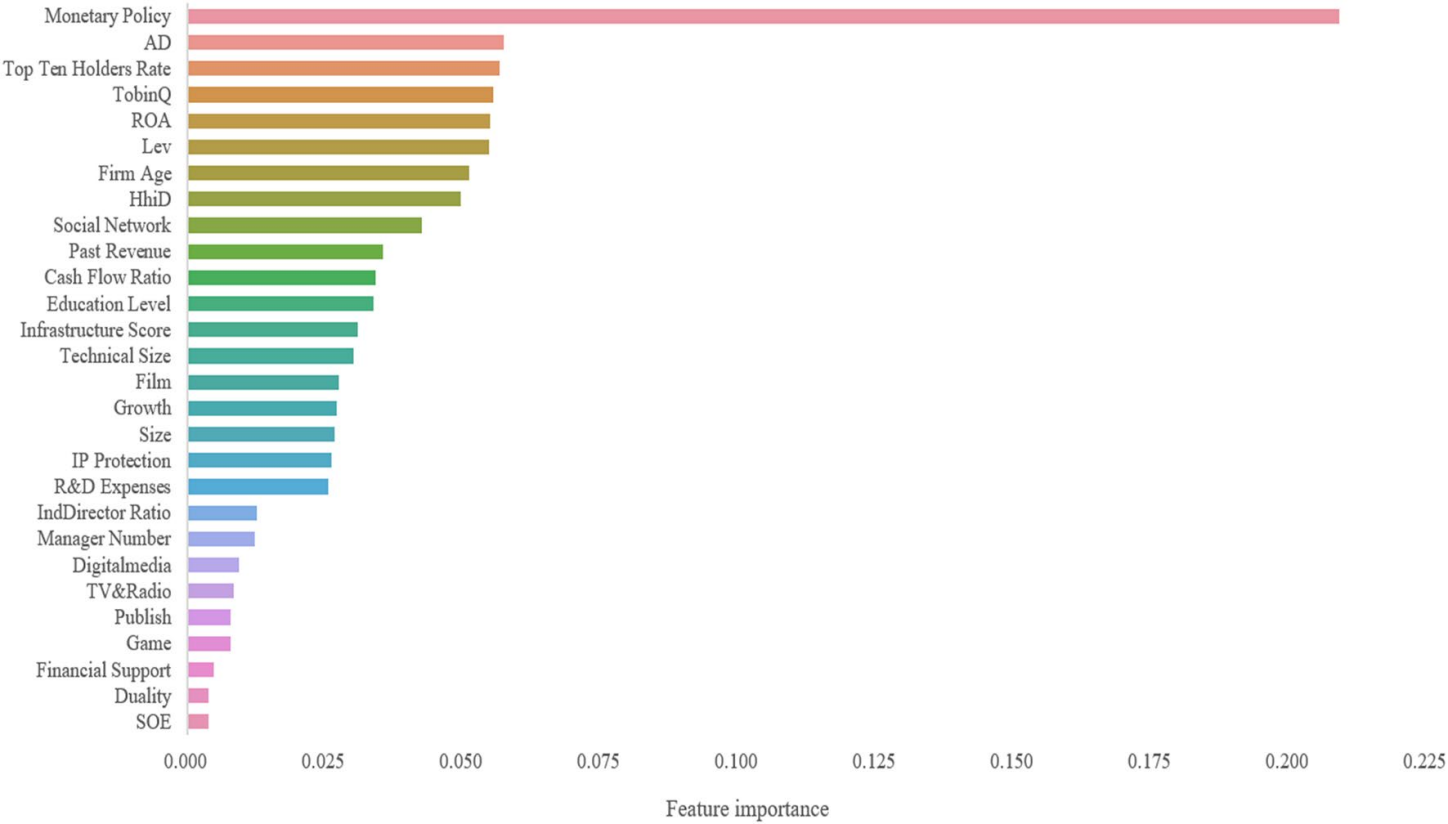
Relative importance ranking based on RFR.

Based on the GBR and RFR, the relative importance of variables in the machine learning model is clear. Figures 2 and 3 report the relative importance ranking of the variables. Table 11 shows the variables ranked in the top 15 of the GBR and RFR methods, indicating that these characteristics are the key elements affecting the digital transformation of Chinese media companies.

**Table 11:** Order of relative importance (top 15).

GBR				RFR			
Rank	Feature	Dimension	Feature importance	Rank	Feature	Dimension	Feature importance
1	Monetary policy	E	0.22037932	1	Monetary Policy	E	0.20904869
2	Top ten holders rate	O	0.06949891	2	AD	I	0.05735817
3	TobinQ	O	0.0636386	3	Top ten holders rate	O	0.05659341
4	HhiD	E	0.06146551	4	TobinQ	O	0.05559654
5	AD	I	0.06044277	5	ROA	O	0.0549122
6	ROA	O	0.04636747	6	Lev	O	0.05469888
7	Film	I	0.04552869	7	Firm Age	I	0.0511105
8	Social network	O	0.0439056	8	HhiD	E	0.04952929
9	Lev	O	0.0395764	9	Social network	O	0.04253711
10	Firm age	I	0.03814815	10	Past revenue	Benchmark	0.03549404
11	Cash flow ratio	Benchmark	0.03723253	11	Cash flow ratio	Benchmark	0.03423758
12	Infrastructure score	E	0.03629936	12	Education Level	O	0.03379038
13	Technical size	T	0.03490361	13	Infrastructure score	E	0.03081521
14	Game	I	0.02863985	14	Technical size	T	0.03017001
15	Education level	O	0.02791289	15	Film	I	0.02747689

#### Prediction model of digital transformation of media companies by important driving factors

**Figure 4:**
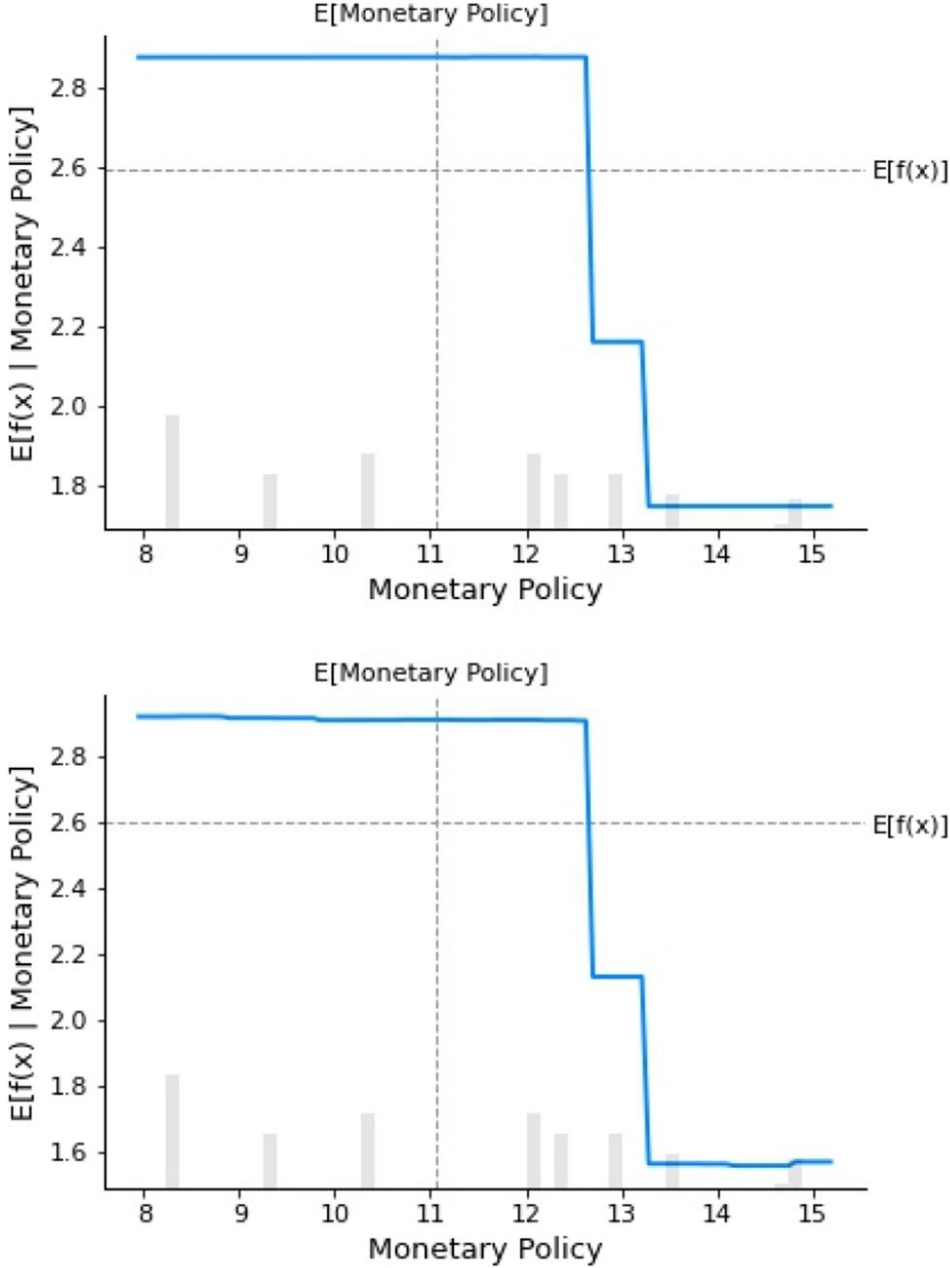
Partial dependence on monetary policy.

**Figure 5:**
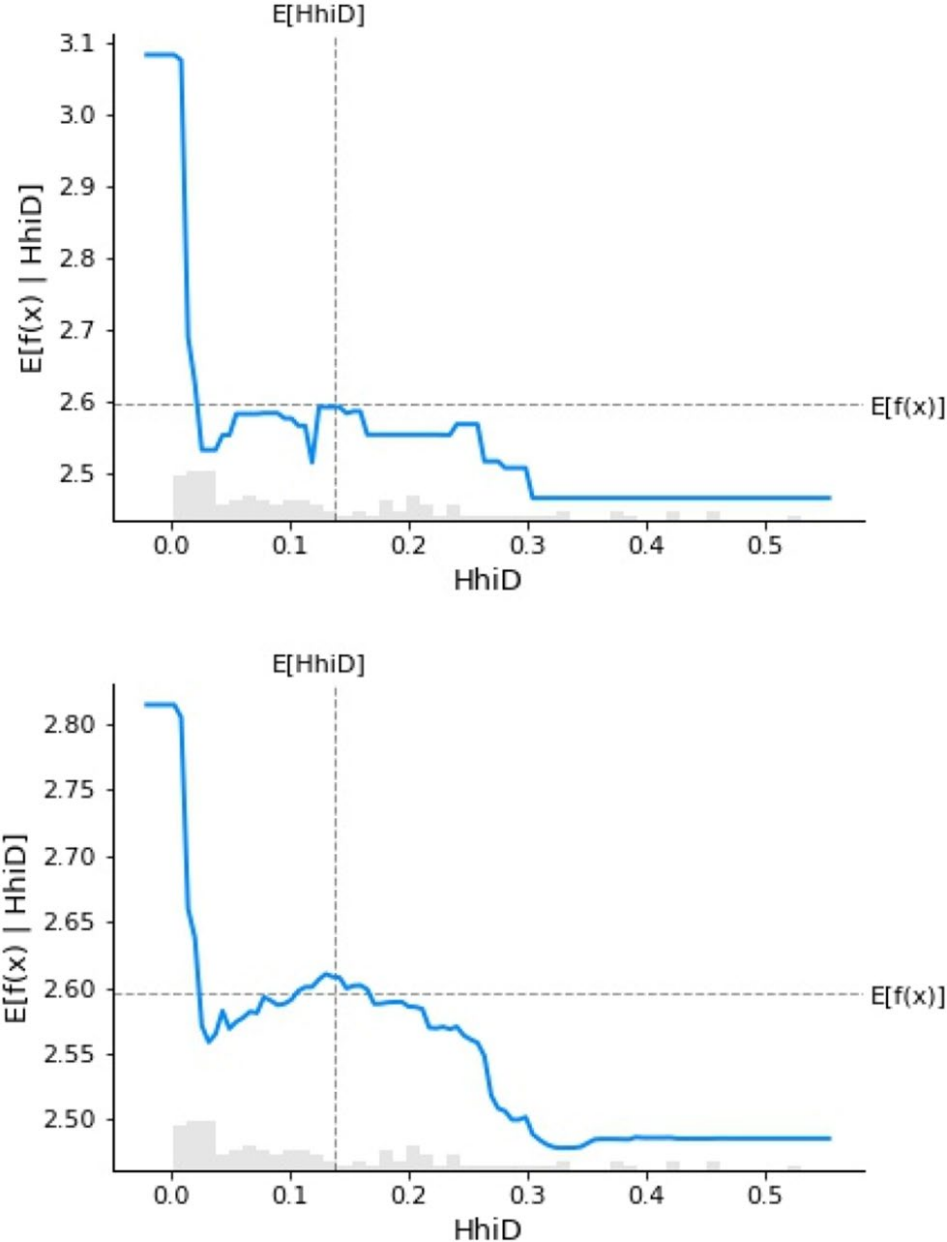
Partial dependence chart of industry competitiveness.

**Figure 6:**
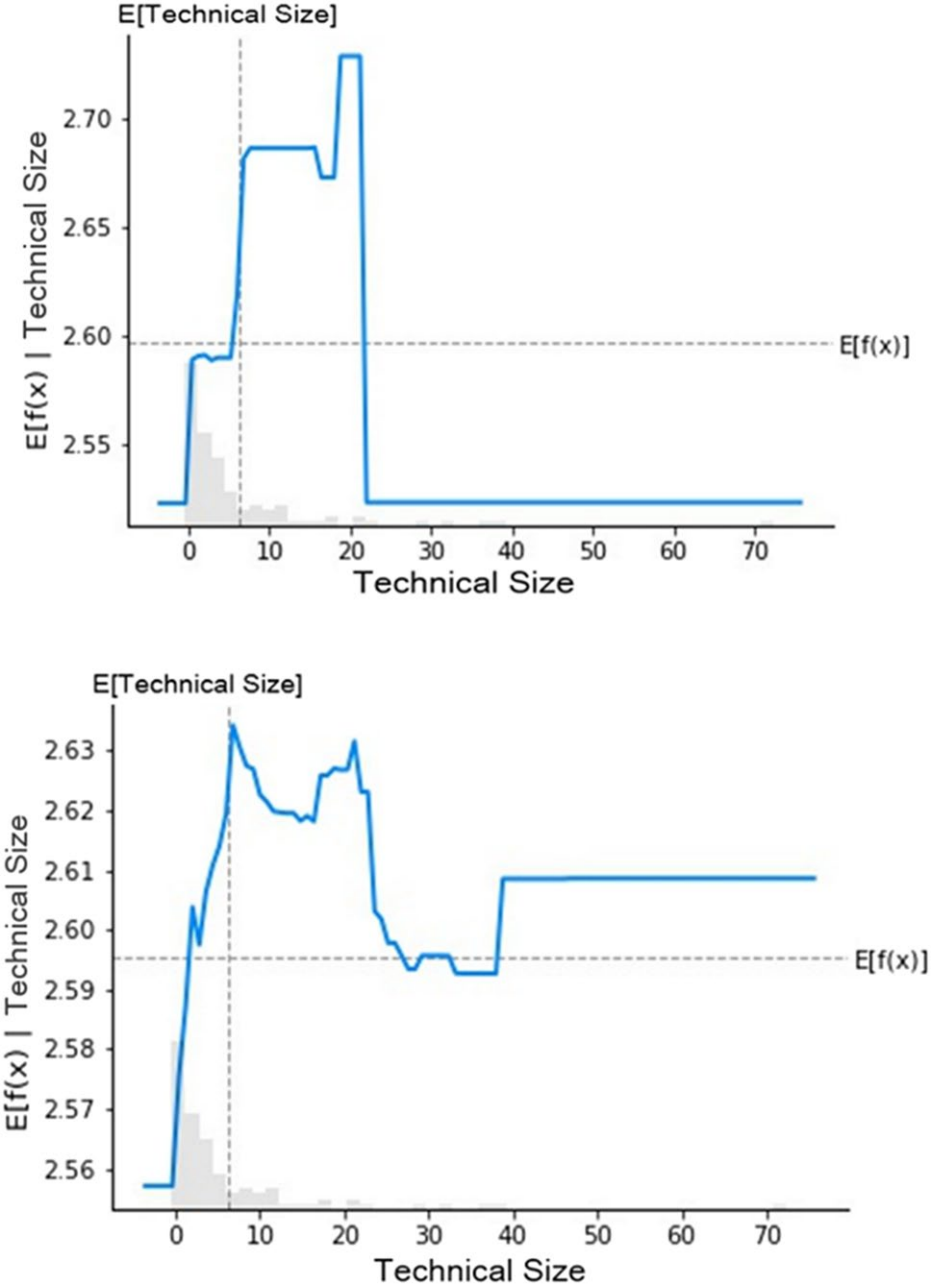
Partial dependence of technical size.

**Figure 7:**
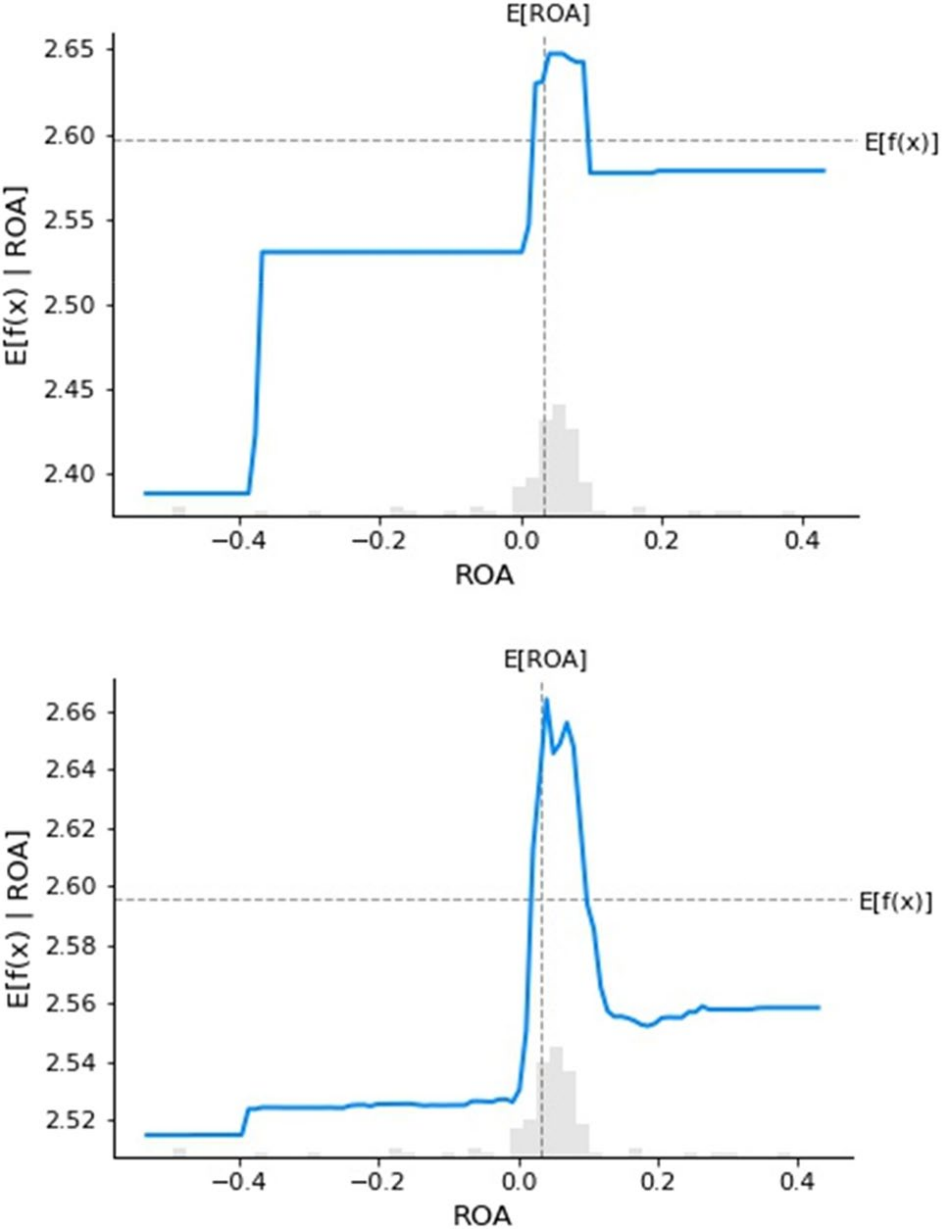
Partial dependency of the ROA.

Following the prediction method of GBR and RFR (The order in Figures 4–7 is as follows), among the many factors that affect the digital transformation of media companies, this study found that monetary policy, industry competition pressure, the proportion of technical size, return on assets of enterprises, age of the listed companies and industry classification have the best effect on predicting the digital transformation of media companies.

##### Monetary policy

Figure 4 is a partial dependency graph of monetary policy. The agent variable of monetary policy is the growth rate of M2. As shown in the figure, when the growth rate of M2 is less than 12.5%, there is no obvious impact on the degree of digital transformation of enterprises. However, when the growth rate is higher than 12.5%, the degree of digital transformation in enterprises shows a downward trend. Therefore, this study holds that the impact of monetary policy on the digital transformation of enterprises is not monotonous, and that enterprise managers should pay attention to the external environment at all times and adjust the process of the digital transformation of media companies timely.

##### Industry competition pressure

Figure 5 shows the HhiD of the industry as a tool to measure the level of competition among companies. Industry competition reflects the intensity in the competition for limited resources among companies. When the index is less than 0.05%, its impact on the digital transformation of media companies is significant and monotonous. When the index is 0.05% − 0.3%, the degree of digital transformation of media companies slowly decreases under its action, and when it reaches 0.3%, the impact effect is small. This shows that when the HhiD is high, media companies have low entry barriers and stable profit flow, thus enterprises have little or no demand to achieve differentiation in homogeneous competition.

##### The proportion of technical size

Figure 6 shows a partial dependence diagram of the proportion of technical size. This study selected the proportion of technical size as the agent variable. When the proportion of technical size is less than 10%, its influence on the digital transformation index of media companies shows a rapid increasing trend. When the proportion approaches 20%, the impact on the digital transformation index is highest. Above this, the impact effect is significantly reduced. Therefore, media companies should determine the proportion of technical size according to the requirements of digital transformation.

##### Return on assets of enterprises

Figure 7 shows a partial dependency graph of the return on assets of an enterprise. As shown, when the ROA of a media company is negative, this indicates problems in the operation of the enterprise. Managers will put more time and energy into business activities, rather than focusing on digital transformation, so the impact is small. When the return on equity of media companies is positive, this shows a temporary trend of rapid increase, followed by stability, indicating that its impact on the intensity of digital transformation is fluctuating.

##### Age of the listed companies

**Figure 8:**
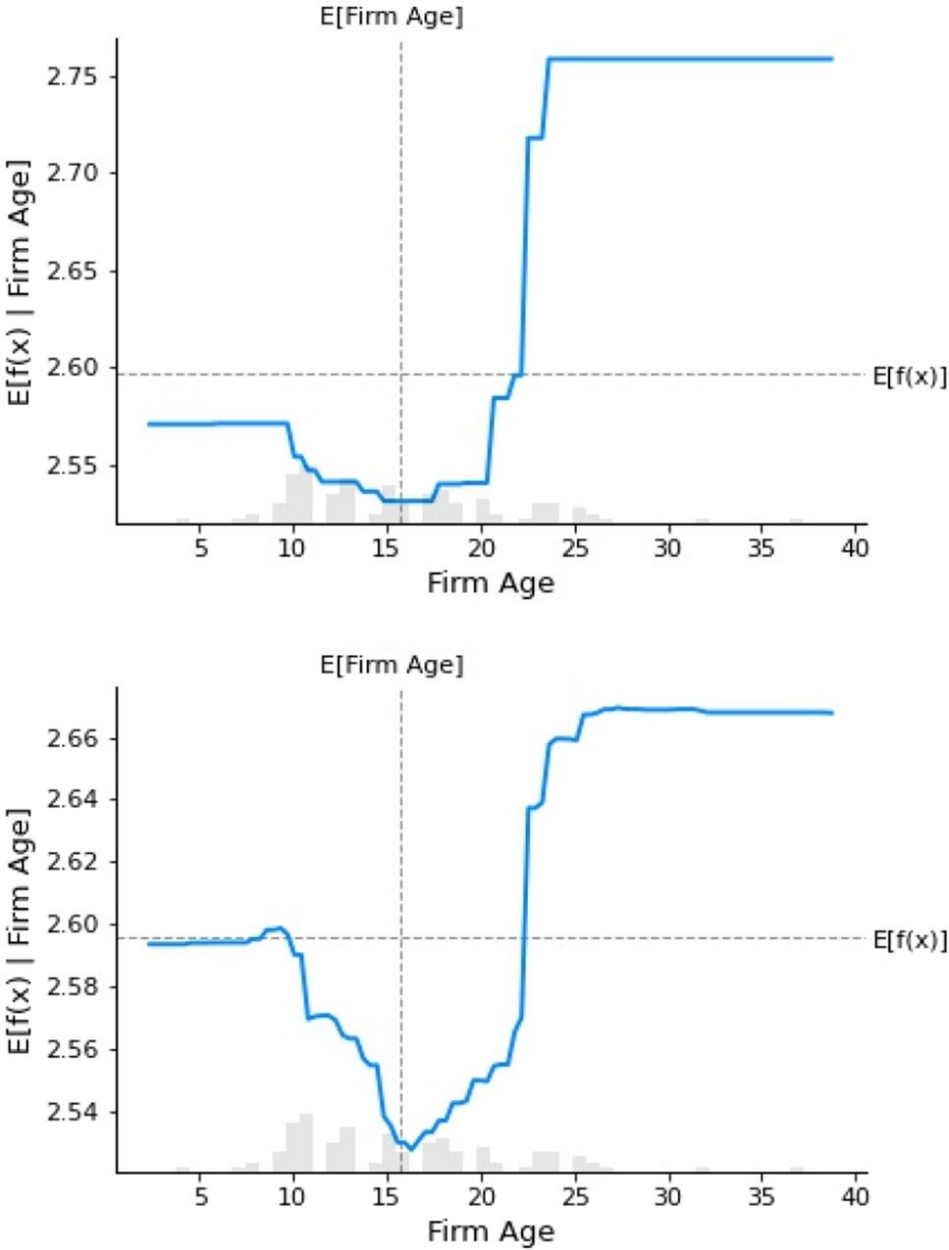
Partial dependence on the age of listed companies.

Figure 8 shows the age of the listed company, used to describe the companies’ characteristics in the benchmark variables. The results indicated that when the establishment period of a media company is less than 15 years, the impact on the digital transformation of companies is not significant. When the establishment period of a media company reaches 15 years, the effect on the digital transformation is minimal, and then shows a significant increasing trend until the establishment period reaches 25 years. Media company management experience affects the strength of the digital transformation. When the company age is small, and lacks operating experience, the digital transformation is relatively low. In contrast, established companies with greater ability and resources can effectively support the digital transformation reform process, making full use of information advantage and achieving scale effects.

##### Industry classification

This research innovatively integrates the dimension of the industry driving force using the theoretical framework of TOE and forms the “TOE-I” model to predict the intensity of digital transformation of Chinese media companies. Firstly, through the two integrated machine learning methods of GBR and RFR, the model is shown to have a significant prediction effect on the advertising industry and the film and television industry, indicating that “TOE-I” can be better applied to the digital transformation prediction of the advertising industry and the film and television industry. Secondly, the prediction ability of radio and television, digital media, and the publishing industry is weak. As traditional mainstream media, radio, television and publishing media extend China’s mainstream media to the Internet field, the stronger the political attribute, the stronger the uncertainty of digital transformation, and the more difficult it is to accurately predict using the “TOE-I” model. Thirdly, game companies have usually been established more recently and are based on digital technology, so taking the digital transformation index as the measurement standard, the effect of digital transformation prediction is not significant enough. See Table 12 for details.

**Table 12:** Comparison of industry factors on digital transformation of media companies.

Industry	GBR	RFR
AD	0.06044277	0.05735817
Film	0.04552869	0.02747689
Game	0.02863985	0.00788905
Publishing	0.01852726	0.00790305
Digitalmedia	0.01667951	0.00938873
TV & radio	0	0.00843955

##### Robustness test

First, changing the training set division method. This study used the 8:2 proportion random classification to determine the training set and the test set, which weakens the randomness to some extent. Therefore, the K-fold method was used for further random division in the robustness test. In machine learning, K-fold cross-validation is a common method of model evaluation. It can help us accurately evaluate the performance of machine learning models and provides more reliable results particularly if the data is limited. The validation steps of the K-fold method were as follows:

- The original dataset was split randomly into K subsets of similar size, taking K values of 10.
- One subset was selected as the validation set and the remaining K-1 subset as the training set.
- The model was trained using the training set and evaluated on the validation set.
- Steps 2 and 3 were repeated until each subset is used as a validation set.
- The results of K times of evaluation were integrated to obtain the final model evaluation index.

Based on this, K-fold cross validation can be repeated through the process of more stable evaluation results to reduce the contingency caused by different data divisions. For small data sets, K-fold cross validation can better evaluate the performance of the model, reducing the data caused by overfitting or underfitting problems.

As shown in Table 13, after replacing the training and test sets using the K-fold test, the correlation findings are compared to Table 9 with no change.

**Table 13:** Test of robustness-Panel A.

	${\text{R}}_{\text{Is}}^{2}$ (1)	${\text{R}}_{\text{oos}}^{2}$ (2)	${\text{EVS}}_{\text{oos}}$ (3)	${\text{MSE}}_{\text{oos}}$ (4)	${\text{MAE}}_{\text{oos}}$ (5)	${\text{MedAE}}_{\text{oos}}$ (6)
Multiple linear regression	0.39732738	0.25957264	0.76428389	0.26846889	0.948616	0.644222
LASSO	0.05365541	0.01575251	0.8751177	0.03963723	1.25559015	0.69827659
GBR	0.9090655	0.41838766	0.6334473	0.42640994	0.72991383	0.45977579
RFR	0.93187938	0.54534248	0.57231571	0.55658528	0.58650539	0.45247681

Second, changing the measure of the intensity of digital transformation. Drawing on Xiao^61^, this study used different entries of enterprise digital transformation strength, eliminated the term “digital technology application” at the application level, and only retained the terms “artificial intelligence”, “blockchain”, “cloud computing”, and “big data” at the basic digital technology level. Add 1 to the total occurrence frequency and take the natural logarithm as the replacement variable for robustness testing. Using new response variables and refitting the model, the results were consistent with the main test, and the specific tests are shown in Table 14.

**Table 14:** Test of robustness-Panel B.

	${\text{R}}_{\text{Is}}^{2}$ (1)	${\text{R}}_{\text{oos}}^{2}$ (2)	${\text{EVS}}_{\text{oos}}$ (3)	${\text{MSE}}_{\text{oos}}$ (4)	${\text{MAE}}_{\text{oos}}$ (5)	${\text{MedAE}}_{\text{oos}}$ (6)
Multiple linear regression	0.3734169	0.40623626	0.40764222	5.44285349	48.6353939	4.69671562
LASSO	0.1554631	0.13122528	0.14218973	6.66321814	71.1616389	5.1286394
GBR	0.91436809	0.52840738	0.52843259	4.72144309	38.628315	3.60535037
RFR	0.92155042	0.54736891	0.54820743	4.81261522	37.07517	4.137019

## Conclusion

Previous research has focused on the correlation between a single factor of a single dimension feature and the digital transformation of media industry, only conducting predictions within the sample, and lacking a comprehensive consideration of the driving forces in the digital transformation of media companies^5–7^. In this study, the driving forces of the digital transformation of Chinese media companies are divided into four dimensions: technology, organization, environmental, and industry drivers (i.e., the “TOE-I” model). The purpose of classifying the driving forces of digital transformation is to explore the differences in the prediction ability of media companies concerning digital transformation. This research analyzed the key driving factors of the digital transformation of media companies and the specific mode influenced by the above factors of the digital transformation of the Chinese media industry.

This study innovatively used ensemble learning methods, taking relative importance indicators and partial dependency graphs to help realize the research purpose. By comparing the fitting effect of the combination of different dimensions of driving forces in the benchmark model, we found that the environmental driving force can predict the digital transformation behavior of the Chinese media industry effectively and accurately, showing environmental drivers to be the dominant factor in influencing Chinese media companies’ strategies for digital transformation. Compared to linear methods such as multiple linear regression, the ensemble learning method achieved better performance in both model interpretation ability and minimization of prediction error, with the RFR method having the best predictive performance. The driving factors of (1) monetary policy, competition pressure in the industry, and the infrastructure index in the environmental driving force, (2) the equity concentration, enterprise value, executive team knowledge level and social networks in the organizational driving force, and (3) advertising, film and television industries in the industry driving forces all have significant predictive effects on the digital transformation of media industry in China.

Based on the above conclusions, the following policy implications are suggested:

1. Policy makers are supposed to provide stable monetary policies. Media companies like game companies and Internet advertising and marketing companies that think globally should be provided with stable economic and financial policies to facilitate their digital transformation. As shown in Fig. 4, the intensity of digital transformation is highest when the M2 growth rate is 12.5%. Therefore, government managers should provide a stable monetary policy to promote the digital transformation of media companies. Moreover, as demonstrated by the prediction ability of the digital transformation, the dimension of the external environmental driving force has the greatest impact on the digital transformation of media companies. Therefore, in addition to providing a stable monetary policy, competition between industries should be guided to provide the matching infrastructure conditions for digital transformation.
2. Managers should maintain stable profit sources to promote digital transformation in media companies; for example for companies in radio, television, and newspapers with strong political attributes and little focus on income output and income. This study indicates that ensuring a positive cash flow of enterprises is an important driving factor for the digital transformation. Attention should also be paid to the decision-making role of core managers in the process of digital transformation. This research adopted two machine learning prediction methods—GBR and RFR that showed enterprise core managers to be an important influence in the prediction factors of China media companies (refer to ranking 2 of GBR and ranking 3 of RFR in Table 11). Thus, to ensure digital transformation, media companies should focus on the decision-making role of core managers. Furthermore, media companies should pay attention to the cultivation and preservation of digital technical talent. In the gradual conversion from traditional content and news production to digital, platform-based production, the cultivation and preservation of technical talent are crucial. Figure 6 illustrates that when the proportion of technical personnel in the company is 20%, digital transformation is the highest. Therefore, media companies should recruit digital technical personnel to maintain this level.
3. Within the media industry, it is necessary to seize the opportunity of technological change and pay close attention to policy changes. As shown in this study, the establishment of enterprises, the change of media, and the internal gap in the media industry all cause differences in the digital transformation of the media industry.
4. There is a gap in the application of empirical research and machine learning methods in existing media economics research, so the application of machine learning in the fields of media economics, journalism, and communication should be continuously promoted.

## Data Availability

The data that support the findings of this study are available from the corresponding author upon reasonable request.

## References

[1] Cui B, Yu H (2023). China media industry development report 2023. Media..

[2] Cui B, Zhao M, Ding M (2023). Media Blue Book: China Media Industry Development Report.

[3] Yuan YP (2023). Government digital transformation: Understanding the role of government social media. Govern. Inf. Q..

[4] Waisbord S (2023). Continuities and breaks in digital journalism and media systems. Dig. Journal..

[5] Xie Y, Li B (2022). Evaluation of competitiveness portfolio of listed media groups in the big data era. Journal. Commun. Rev..

[6] Pan A, Wang X (2023). How does digital transformation promote the high-quality development of cultural enterprises. J. Shenzhen Univ..

[7] Xing M, Chen D (2023). How does digital transformation leverage the performance of listed cultural companies? Based on “cost reduction and efficiency increasing” perspective. Cult. Ind. Res..

[8] Kleinberg J, Ludwig J, Mullainathan S, Obermeyer Z (2015). Prediction policy problems. Am. Econ. Rev..

[9] Li Y, Qian Y, Li Q, Li L (2023). Evaluation of smart city construction and optimization of city brand model under neural networks. Comput. Sci. Inf. Syst..

[10] Zhang Z (2022). The theoretical framework, practical logic and realization path of cultural industry digitization. Soc. Sci. Front..

[11] Han X, Guo W, Shi L (2022). Dimension framework and practice strategy of media innovation degree: From the perspectives of content production, market and technological innovation. Contemp. Commun..

[12] Zhang Z (2022). Research on business model innovation mechanism of cultural enterprises based on grounded theory. Theory J..

[13] Verhoef PC (2021). Digital transformation: A multidisciplinary reflection and research agenda. J. Bus. Res..

[14] Yan L, Ling X, Wang Z, Xu Y (2023). Can mixed-ownership reform boost the digital transformation of state-owned enterprises?. Econ. Anal. Policy..

[15] Kraus S (2021). Digital transformation: An overview of the current state of the art of research. SAGE Open..

[16] Roman A, Rusu VD (2022). Digital technologies and the performance of small and medium enterprises. Stud. Bus. Econ..

[17] Zeng H, Ran H, Zhou Q, Jin Y, Cheng X (2022). The financial effect of firm digitalization: Evidence from China. Technol. Forecast. Soc. Chang..

[18] Wu K, Lu Y (2023). Corporate digital transformation and financialization: Evidence from Chinese listed firms. Financ. Res. Lett..

[19] Tu W, He J (2023). Can digital transformation facilitate firms’ M & A: Empirical discovery based on machine learning. Emerg. Mark. Financ. Trade..

[20] Lange F, Tomini N, Brinkmann F, Kanbach DK, Kraus S (2023). Demystifying massive and rapid business scaling: An explorative study on driving factors in digital start-ups. Technol. Forecast. Soc. Chang..

[21] Ardolino M (2018). The role of digital technologies for the service transformation of industrial companies. Int. J. Prod. Res..

[22] Yu G, Liu Y (2023). Understanding generative Al: An examination of a milestone in the history of the internet's development. Media Observ..

[23] Huang H (2022). Research on the effect mechanism of Chinese internet platform company's financialization and capital expansion. Media Econ. Manag. Res..

[24] Sheng H, Zhang J, Zhang K (2022). The characteristics and performance of M & A of press and publication media enterprises: Configuration analysis of fs OCA. Journal. Res..

[25] Guzman AL, Lewis SC (2020). Artificial intelligence and communication: A human–machine communication research agenda. New Media Soc..

[26] Opdahl AL (2023). Trustworthy journalism through AI. Data Knowl. Eng..

[27] Goldani MH, Safabakhsh R, Momtazi S (2021). Convolutional neural network with margin loss for fake news detection. Inf. Process. Manag..

[28] Duan HK, Vasarhelyi MA, Codesso M, Alzamil Z (2022). Enhancing the government accounting information systems using social media information: An application of text mining and machine learning. Int. J. Account. Inf. Syst..

[29] Wang J, Liu YL (2023). Deep learning-based social media mining for user experience analysis: A case study of smart home products. Technol. Soc..

[30] Shi B, Wang H (2022). An AI-enabled approach for improving advertising identification and promotion in social networks. Technol. Forecast. Soc. Chang..

[31] Sun J, Li N, Zhao M (2023). Analysis and machine learning negative media coverage and corporate financial distress prediction: Based on text. Collect. Essays Financ. Econ..

[32] Tornatzky, L. G. & Fleischer, M. The processes of technological innovation. Lexington, MA: Lexington Books (1990).

[33] Awa HO, Ojiabo OU, Orokor LE (2017). Integrated technology organization-environment (T-O-E) taxonomies for technology adoption. J. Enterpr. Inf. Manag..

[34] Muchenje G, Seppanen M (2023). Unpacking task-technology fit to explore the business value of big data analytics. Int. J. Inf. Manag..

[35] Delke V, Schiele H, Buchholz W, Kelly S (2023). Implementing Industry 40 technologies: Future roles in purchasing and supply management. Technol. Forecast. Soc. Change..

[36] Hadwer AA, Tavana M, Gillis D, Rezania D (2021). A systematic review of organizational factors impacting cloud-based technology adoption using technology-organization-environment framework. Internet Things..

[37] Shi X, Zhang Y, Wu Y, Wu H (2023). Political turnover and firm innovation in China: The moderating role of innovation and entrepreneurship environment. J. Asian Econ..

[38] Luo Y, Cui H, Zhong H, Wei C (2023). Business environment and enterprise digital transformation. Financ. Res. Lett..

[39] Yang J, Xu N (2023). Research on the influence of dynamic capability and top managerial social capital on the digital transformation of enterprises. J. Technol. Econ..

[40] Zhang M, Wang D, Zeng N, Fu S (2023). How to become a sturdy grass withstanding strong wind? A study on the antecedent configurations of entrepreneurial ecosystem resilience based on WSR. Methodol. Manag. Rev..

[41] Chen P, Hao Y (2022). Digital transformation and corporate environmental performance: The moderating role of board characteristics. Corpor. Soc. Respons. Environ. Manag..

[42] Yu G, Fang K (2020). Will algorithmic content delivery result in information cocoons? A positive analysis based on media diversity and trust in information source. Shandong Soc. Sci..

[43] Nie P (2021). Prediction of home energy consumption based on gradient boosting regression tree. Energy Rep..

[44] Parzinger M (2022). Comparison of different training data sets from simulation and experimental measurement with artificial users for occupancy detection: Using machine learning methods Random Forest and LASSO. Build. Environ..

[45] Chen CH, Tanaka K, Kotera M, Funatsu K (2020). Comparison and improvement of the predictability and interpretability with ensemble learning models in QSPR applications. J. Cheminform..

[46] Ghazwani M, Begum MY (2023). Computational intelligence modeling of hyoscine drug solubility and solvent density in supercritical processing: Gradient boosting, extra trees, and random forest models. Sci. Rep..

[47] Chen X, Cho YH, Dou YW, Lev B (2022). Predicting future earnings changes using machine learning and detailed financial data. J. Account. Res..

[48] Aceña V, de Diego IM, Fernández R, Moguerza JM (2022). Minimally overfitted learners: A general framework for ensemble learning. Knowl. Based Syst..

[49] Supsermpol P, Huynh VM, Thajchayapong S, Chiadamron N (2023). Predicting financial performance for listed companies in Thailand during the transition period: A class-based approach using logistic regression and random forest algorithm. J. Open Innov..

[50] Friedman JH (2001). Greedy function approximation: A gradient boosting machine. Ann. Statist..

[51] Bernile G, Bhagwat V, Rau PR (2017). What doesn't kill you will only make you more risk-loving: Early-life disasters and CEO behavior. J. Financ..

[52] Schoar A, Zuo L (2017). Shaped by booms and busts: How the economy impacts CEO careers and management styles. Rev. Financ. Stud..

[53] Bandiera O (2020). CEO behavior and firm performance. J. Polit. Econ..

[54] Xu N, Yuan Q, Jiang X, Kam KC (2015). Founder's political connections, second generation involvement, and family firm performance: Evidence from China. J. Corpor. Financ..

[55] Sun Z, Zheng Y (2021). City government micro-blogging development and driving factors in china based on the combination of big data and small data analysis for 228 cities (2011–2017). J. Public Manag..

[56] Wu Q, Ma X (2021). Analysis on the efficiency of coordinated poverty reduction of government finance and finance supporting agriculture under the background of rural revitalization: Based on our country's inter-provincial panel data. Modern. Manag..

[57] Li S, Li X, Wang S, Tong Y (2023). Family firm succession and digital transformation: Promotion or inhibition?. J. Manag. World.

[58] Li H, Long H, Wu F (2021). Heterogeneous institutional investors and enterprise digital transformation. Financ. Forum..

[59] Zhao X, Chen Q, Zhang H (2023). Firm investment and financial autonomy: A transaction cost economics and firm lifecycle approach. Manag. Decis. Econ..

[60] Hanelt A, Bohnsack R, Marz D, Antunes C (2021). A systematic review of the literature on digital transformation: Insights and implications for strategy and organizational change. J. Manag. Stud..

[61] Xiao T, Wu Y, Qi W (2022). Does digital transformation help high-quality development of enterprises? Evidences from corporate innovation. Bus. Manag. J..

